# Skin Sensitization Testing—What’s Next?

**DOI:** 10.3390/ijms20030666

**Published:** 2019-02-04

**Authors:** Gunilla Grundström, Carl A.K. Borrebaeck

**Affiliations:** 1 SenzaGen AB, Medicon Village, S-223 81 Lund, Sweden; gunilla.grundstrom@senzagen.com; 2 Department of Immunotechnology, Lund University, Medicon Village (bldg 406), S-223 81 Lund, Sweden

**Keywords:** genomics, machine learning, skin sensitization, adverse outcome pathways, next generation in vitro tests

## Abstract

There is an increasing demand for alternative in vitro methods to replace animal testing, and, to succeed, new methods are required to be at least as accurate as existing in vivo tests. However, skin sensitization is a complex process requiring coordinated and tightly regulated interactions between a variety of cells and molecules. Consequently, there is considerable difficulty in reproducing this level of biological complexity in vitro, and as a result the development of non-animal methods has posed a major challenge. However, with the use of a relevant biological system, the high information content of whole genome expression, and comprehensive bioinformatics, assays for most complex biological processes can be achieved. We propose that the Genomic Allergen Rapid Detection (GARD™) assay, developed to create a holistic data-driven in vitro model with high informational content, could be such an example. Based on the genomic expression of a mature human dendritic cell line and state-of-the-art machine learning techniques, GARD™ can today accurately predict skin sensitizers and correctly categorize skin sensitizing potency. Consequently, by utilizing advanced processing tools in combination with high information genomic or proteomic data, we can take the next step toward alternative methods with the same predictive accuracy as today’s in vivo methods—and beyond.

## 1. Introduction

The support and demand for developing accurate, non-animal alternative methods for safety assessment have been the top priority for scientists and regulatory authorities for many years. The factors driving the investments in developing non-animal methods include ethical considerations, societal expectations, legislative change, and a general desire to exploit the opportunities provided by new scientific abilities, such as genomics, deep learning, and an improved understanding of the immune system. In the area of skin sensitization, the investments have been particularly significant, and many alternative test methods for hazard identification have been developed. However, most methods rely on the interrogation of a single endpoint in a very complex and multi-faceted biological pathway that results in skin sensitization. It can be argued that the next stage in the evolution of non-animal methods for the assessment of hazards will require a more holistic evaluation of the acquisition of skin sensitization, as has been the trend in other complex diseases.

Skin sensitization has been described in an adverse outcome pathway (AOP) with defined key events (KEs) aiming to increase the mechanistic understanding and interpretation of data and aid in the development of reliable tests [1]. The focus has initially been on the development of in vitro methods based on these key events and has resulted in a handful of in chemico and in vitro tests that have been assigned a test guideline by the Organization for Economic Co-operation and Development (OECD). Each of these validated assays (Direct Peptide Reactivity Assay (DPRA), human Cell Line Activation Test (h-CLAT), KeratinoSens™, U-SENS™, LuSens, and IL-18 Luc) are single-point tests, yielding very limited if any mechanistic insight; therefore, neither any one of them nor any other test has been recognized or proposed as a possible standalone assay to replace the golden standard, e.g. the murine LLNA (Local Lymph Node Assay). 

However, skin sensitization is the result of multi-mechanistic molecular and cellular events. No single endpoint contains enough information to mimic such a complexity in vitro, and the development of accurate in vitro test methods must be based on multi-parametric analysis, as is evident from other complex disease areas [2]. To overcome the lack of comprehensive alternative non-animal tests, integrated strategies to increase accuracy and sensitivity by different ways of combining existing tests are now proposed in a new guideline on defined approaches for skin sensitization. Even though such integrated strategies, largely based on weight of evidence, may somewhat improve the performance of today’s established tests, in certain circumstances the combination of individual tests can actually result in a worse outcome in terms of test accuracy [3,4]. The question is rather whether it is time to introduce conceptual novel approaches, such as genomics-based and data-driven test principles, to continue to drive the evolution of test strategies that better reflect the underlying biological complexity.

## 2. Genomics and Deep Learning—Data-Driven Progression of Alternative Test Methods

Since skin sensitization is a complex process requiring the coordinated and tightly regulated interaction between a variety of cells and molecules, one approach would be to create a genetic biomarker map of these events. The entire human genome consists of over 20,000 coding genes, which contain the information that regulates all cellular processes and can be considered a blueprint of our biological existence. How many of, and to what extent, these genes are specifically involved in the immune response to a chemical sensitizer has not been fully explored. However, with recent achievements in artificial intelligence and neuronal networks, including supervised and unsupervised machine learning techniques, vastly complex data sets such as whole genome transcripts can be processed into highly accurate and interpretable read-outs.

Machine learning is not new to the field of life science and has been successfully applied in both genetics, e.g., the identification of disease-associated genes and genetic biomarkers [5], and medicine, e.g., the automated interpretation of electrocardiogram, the detection of a lung nodule from a chest X-ray, and the Framingham Risk Score developed for coronary heart disease [6]. Within the field of chemical risk assessment, machine learning has been successfully applied in the development of QSAR (quantitative structure–activity relationship) approaches for skin sensitization potential [7,8,9]. While QSARs are powerful predictive tools, they are based on an existing mechanistic understanding and are not intended to further our understanding of underlying mechanisms.

Next-generation sequencing technologies have enabled clinical application of diagnostic tests based on whole genome analysis, offering possibilities to accurately diagnose a variety of diseases and aid in the development of specific targeted therapies. Similarly, genomics-based methods have the potential to fully explore the complex mechanistic information derived from the transcriptome analysis of cells/tissues/organs, after challenge by a foreign substance. By translating these genomic signals into predictive models, analytical tests could be developed for a variety of readouts, including decision values for hazard determination and sensitizing potency of substances. Genomic based tests would consequently allow for complex mechanistic analysis of most underlying events of the proposed adverse outcome pathways. With a large enough biological data set from a relevant biological system and adequate machine learning algorithms, an unbiased and a more comprehensive and holistic analysis of the biological specified process, such as skin sensitization, can be achieved.

The GARD™ (Genomic Allergen Rapid Detection) assay was developed with the aim to create a data-driven, scientifically valid in vitro model of skin sensitization with a high informational content to reflect the complex processes underlying the immune response. A whole genome expression of a human dendritic cell line, a central cellular orchestrator of adaptive immune responses and known to play pivotal roles in the acquisition of skin sensitization, is analyzed after exposure to a sensitizing agent to create a blueprint of the human in vivo reaction. The transcriptome is then submitted to pattern recognition and machine learning techniques to develop a specific prediction model of the sensitizing potential. The resulting skin sensitization test is based on a biomarker signature, consisting of nearly 200 genes representing the immunological response of dendritic cells to the allergenic moiety itself. The assay has been shown to have an accuracy of over 90%, based on more than 100 tested chemicals [10,11,12], and similar models for predicting potency classification of skin sensitizers and respiratory sensitization have also been developed [13,14].

## 3. Heading for the Next Generation of Sensitization Tests

As mentioned above, there is an increased regulatory and societal demand for alternative in vitro skin irritation and sensitization test systems, which is reflected in the ban of animal testing of cosmetic ingredients in 2013 and the ongoing revision of the international standard for Biological Evaluation of Medical Devices (ISO 10993-10), where in vitro testing of both skin irritation and sensitization will be required. Furthermore, in recent decades there has been a progressive investment in addressing the three Rs (replacement, reduction, and refinement) in biomedical sciences, putting even more emphasis on the urgent need for non-animal test alternatives. The ultimate alternative in vitro test would be a highly predictive, accurate and sensitive system that by itself (standalone) could replace the golden standard of today, i.e. LLNA, and identify, and preferably distinguish between, skin and respiratory sensitizers as well as determine sensitizing potency. 

To embrace the complex process of skin sensitization, a standalone in vitro test needs to comprise the coordinated and tightly regulated interactions between different cell types and molecules. Dendritic cells (DCs) undergo both functional and phenotypical changes, after the encounter and recognition of a foreign substance, in order to initiate and orchestrate a full adaptive immune response [12,15,16]. Hence, DCs are relevant cells for the evaluation of chemical sensitizers regardless of whether the chemical exposure occurs in the skin, the respiratory tract, or the gastro-intestinal tract. In addition to DCs, epithelial cells play a crucial role in the sensitization phase by phenotypical changes upon chemical stimulation, resulting in the release of, e.g., cytokines and reactive oxygen species [17,18]. Consequently, both dendritic cells and skin epithelial cells (keratinocytes) of human origin are appropriate candidates for developing relevant in vitro skin sensitization tests. Particularly, if combined with high information content technologies, such as genomics, epigenomics, or proteomics.

The regulatory endorsed in vitro methods are today all based on one out of three generally approved approaches aligned to a specific AOP key event (KE). The first approach reflects the KE1—the haptenation of skin proteins—and evaluates the ability of test chemicals to covalently bind to proteins (DPRA, Direct Peptide Reactivity Assay). The second approach reflects the KE2—the generation of danger signals by keratinocytes—and today exploits the Nrf2-Keap-1-antioxidant response element (ARE) pathway (KeratinoSens and LuSens assays). Finally, the third approach represents the KE3—the activation of dendritic cells measured by the elevated expression of cell surface molecules and inflammatory cytokines—and is today represented by the membrane determinants CD86 and CD54 (h-CLAT and U-SENS) and the release of IL-8 for integrated testing (IL-8 Luc). Since the AOP of skin sensitization represents a somewhat oversimplified and non-continuous view, built upon prior existing mechanistic understanding, it does not per se deliver a new mechanistic understanding of the process. 

Hence, our view is that, when developing new predictive models, it is important not to be restrained by existing mechanistic understanding or the prevailing assumption that a test by necessity must address all key events. This point can be illustrated by the development of the LLNA assay [19], which was based on the current understanding of the biological mechanisms, in particular that sensitization involved clonal expansion of T-cells in the lymph node upon antigen presentation (i.e. a model of the final KE4 of today’s AOP) without the need to consider preceding events. Similarly, the first structure–activity relationships (SARs) were based on the recognition that a covalent reaction with proteins leading to the formation of antigens is a prerequisite for sensitization by non-macromolecular chemicals, without further understanding the downstream events [20]. 

The assay GARD^TM^ skin was on the other hand developed, using a biologically relevant system mimicking the immune response, looking unconditionally at changes in gene expression patterns after exposure to known sensitizers. The GARD™ skin biomarker signatures hence represent all relevant genes regardless of whether their function was previously known or considered relevant. Some of the genes are known to be part of the pre-defined key events (KEs) of the adverse outcome pathway (AOP) selected for skin sensitizing, while other mirror established immunological responses, and some yet to be explored. 

To emphasize the strength of a holistic data-driven approach based on a high information content and state-of-the-art data processing techniques, one can elaborate on a pathway analysis of the GARD™ skin biomarker signature. In one single test, genes involved in danger signals, such as (i) oxidative stress responses, (ii) inflammasome complex formation, and (iii) pro-inflammatory cytokine and chemokine signaling, and dendritic cell activation and maturation, (iv) pattern recognition receptors, heat shock proteins, and mitogen-activated protein kinase (MAPK) activation, (v) immunological self-defense mechanisms, (vi) cell migration, and (vii) innate immune system activation and xenobiotic recognition, are measured and weighed against each other [10]. Although the method is based upon expression patterns of selected genes, GARD is easy to perform and requires no more training than do other methods for skin sensitization. Moreover, the assay has a throughput equivalent to other cell-based methods and has been shown to display high levels of inter-laboratory reproducibility. Apart from SENS-IS, which is a 3D reconstituted epidermis-based model with a biomarker panel of 62 genes [21], GARD™ skin is today the only multi-mechanistic in vitro assay under evaluation for an OECD test guideline.

Genomics and machine learning techniques have been available for decades but have had very little impact on chemical safety assessment, something that stands in stark contrast to the impact of big data analysis and machine learning in many other industries. However, modern data processing techniques are now gaining interest and progress in the interpretation and understanding of the nature of skin sensitization. With machine learning being applied to identify atopic dermatitis from electronic health records [22], the development of QSAR models for both sensitizing potential and potency and the development of holistic assays like GARD, we are progressing into the acceptance of next-generation test models with a potential to be regarded as a standalone test. Consequently, by applying advanced experimental models and deep learning approaches, we can enter the 21st century, where the remaining question is rather when these approaches will gain general and regulatory acceptance.

## 4. Summary

In brief, today’s single-point assays are not comprehensive and accurate enough to be considered as standalone skin sensitization tests that can replace animal tests. Consequently, it is of outmost importance that regulatory authorities evaluate novel, wide-ranging technologies that will drive the development forward toward the desired goal of alternative, high-performance in vitro methods. The possibility to extrapolate from the high informational content contained in DNA opens up opportunities for novel test concepts, capable of not only predicting allergenicity but also distinguishing between skin and respiratory sensitizers, predicting the potency of sensitizers, and helping to gain further mechanistic insights of the sensitization process. This multi-layer information cannot be achieved by analyzing one or two biomarkers, representing single key events in a proposed adverse outcome pathway. 

There is now an extraordinary opportunity to progress to multi-mechanistic test strategies with the help of advanced data processing tools in combination with high throughput readouts. This mindset represents a paradigm shift and will also allow for the accurate prediction of toxicological endpoints, such as skin and respiratory sensitization potential in vitro.

## Abbreviations

AOPs	Adverse Outcome Pathways
DC	Dendritic Cell
GARD	Genomic Allergen Rapid Detection
KE	Key Event
